# Over‐the‐counter cough and cold medicines: reported poisonings of children before and after the 2012 and 2020 labelling changes in Australia

**DOI:** 10.5694/mja2.51865

**Published:** 2023-02-21

**Authors:** Abrar Arbaeen, Nial J Wheate, Jared A Brown, Rose Cairns

**Affiliations:** ^1^ The University of Sydney Sydney NSW; ^2^ NSW Poisons Information Centre Children's Hospital at Westmead Sydney NSW

**Keywords:** Pharmacoepidemiology, Toxicology, Medical errors, Common cold

Respiratory tract infections are common in children. Evidence for the efficacy of over‐the‐counter cough and cold medications in young children is limited,^1^, ^2^ but they can lead to severe adverse events, including death.^3^ In Australia, the Therapeutic Goods Administration (TGA) introduced compulsory labelling changes for non‐prescription cough and cold products in 2012 (but not herbal products),^4^ with further changes in 2020 for first generation (sedating) antihistamine‐containing products^5^ (further details: Supporting Information).

We evaluated whether these labelling changes were followed by reductions in reported poisoning exposures. We reviewed calls to the New South Wales Poisons Information Centre (NSWPIC) during 1 January 2010 – 31 December 2021 about children under six years of age exposed to over‐the‐counter cough and cold medications. NSWPIC receives about 50% of the approximately 220 000 calls to Australian poisons information centres each year. We estimated annual percentage changes (APCs) in annual report rate with the regression program, Joinpoint 4.8.0.1, using permutation test model selection (Supporting Information). The study was approved by the Sydney Children's Hospitals Network Human Research Ethics Committee (2021/ETH00165).

A total of 8327 exposures of children under six years of age to non‐prescription cough and cold products were reported during 2010–2021 (Box). Almost all were accidental exposures (4264, 51.2%) or therapeutic errors (3953, 47.5%) (Supporting Information, table 2). The mean annual number of reported exposures declined significantly, from 1067 before to 587 after the 2012 changes (APC, –6.3%; 95% confidence interval [CI], –10.3% to –2.0%), primarily because of the decline during 2010–2014 (APC, –17.3%; 95% CI, –26.5% to –6.9%). For products subject to the 2012 labelling change (ie, non‐herbal preparations only), the declines were more marked (2010–2021: APC, –11.1%; 95% CI, –14.5% to –7.5%; 2010–2014: –21.4%; 95% CI, –29.2% to –12.7%). The largest decline was for exposures to combinations of sedating antihistamines and decongestants (APC, –15.8%; 95% CI, –26.2% to –3.8%). Exposures to formulations of sedating antihistamines without decongestants did not decline significantly across the entire study period, nor after the 2020 labelling changes, although a reduction during 2010–2018 was evident (APC, –6.8%; 95% CI, –9.8% to –3.8%). Reported exposures to herbal preparations increased during 2010–2021 (APC, 25.1%; 95% CI, 16.0–34.9%) (Supporting Information, table 3, figure 1).

Box 1 Exposures of children under six years of age to over‐the‐counter cough and cold products reported to the New South Wales Poisons Information Centre, 2010–2021*

**Figure:**
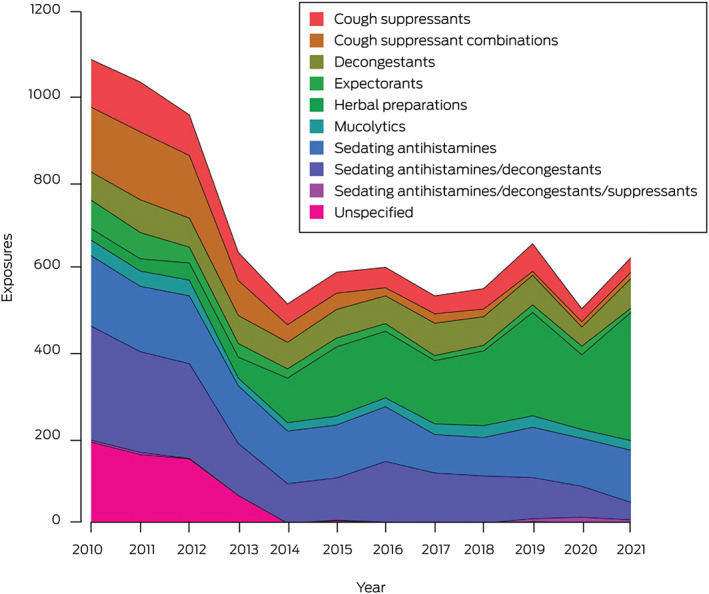
* The raw data for this figure are included in the Supporting Information, table 1.

During the 2018 cold and influenza season (1 April – 31 October), off‐label medication use was involved in 92 of 250 calls (37%) for which this information could be collected, including 54 for brompheniramine/phenylephrine combinations. Off‐label use was recommended by health care professionals in 39 of the 53 cases for which this information was recorded (74%).

The proportions of children exposed to non‐prescription cough and cold products who required hospital management were similar before (334 of 2485, 11.7%) and after August 2012 (686 of 5482, 12.5%), but the mean annual number declined from 125.3 to 73.5 children.

In conclusion, we found that reported poisonings of children under six years of age with non‐prescription cough and cold products have declined since the 2012 labelling changes. In the United States, a substantial decline in the numbers of accidental ingestions and therapeutic errors in children under six years of age followed a similar intervention.^6^ Health care professional and public education campaigns discouraging the use of non‐prescription cough and cold products may have played a role in reducing the number of poisonings in NSW children. Nevertheless, about 600 exposures of children under six, including 75 who require hospital management, are still reported to NSWPIC each year. Further, many herbal products were launched after 2012 as they could be labelled as suitable for young children. These products are generally safe and overdose does not typically cause toxicity, but evidence for their efficacy is very limited.^7^

The continued off‐label use of products for treating coughs and colds in young children suggests that health care professionals and the public may underappreciate their risks. This indicates that further education and clinical guidance are needed.

## Open access

Open access publishing facilitated by The University of Sydney, as part of the Wiley ‐ The University of Sydney agreement via the Council of Australian University Librarians.

## Competing interests

Rose Cairns has an untied educational grant from Reckitt to fund doctoral research concerning over‐the‐counter analgesics, unrelated to the content of this article.

## Supporting information


**Supporting Information**.
