# Supplementary material for: Over‐the‐counter cough and cold medicines: reported poisonings of children before and after the 2012 and 2020 labelling changes in Australia
Source: Med J Aust. 2023 Feb 21;218(9):410–1. doi: 10.5694/mja2.51865 (PMC10953310; doi:10.5694/mja2.51865)
Supplement: Supplementary file 1 — Supporting Information. [file MJA2-218-410-s001.pdf]

The Medical Journal of Australia

Supporting Information

Supplementary methods and results

This appendix was part of the submitted manuscript and has been peer reviewed.
It is posted as supplied by the authors.

Appendix to: Arbaeen A, Wheate NJ, Brown JA, Cairns R. Over-the-counter cough and cold medicines: reported poisonings of children before and after the 2012 and 2020 labelling changes in Australia. *Med J Aust* 2023; doi: 10.5694/mja2.51865.

Labelling changes

The Therapeutic Goods Administration (TGA) introduced compulsory labelling changes to cough and cold medications in 2012. The TGA announced that these medications should not be given to children under the age of 6 years, and should only be given to children aged 6–11 years on the advice of a doctor, pharmacist, or nurse practitioner.[1] Products with new labelling to this effect began to appear in pharmacies in September 2012. New labels do not contain dosing instructions for children under 6 for cough and cold, and use of these medicines in children under 6 now constitutes off-label use. Further labelling changes were introduced in 2020 for first generation (sedating) oral antihistamines, with products required to carry warnings: 'Do not give to children under 2 years of age'.[2]

Supplementary methods

The NSWPIC database was searched for exposures of children to non-prescription cough and cold products, 1 January 2010 – 31 December 2021, by substance codes for the "neonate" (0–4 weeks), "infant" (1–12 months), "toddler" (1–4 years), and "child" (5–14 years) age categories. For this article, report our analysis of data for children under 6 years of age.

Calls with the following substance codes were extracted: "cough and cold preparation: no analgesic", "bromhexine", "brompheniramine", "chlorpheniramine", "dexchlorpheniramine", "diphenhydramine", "doxylamine", "pheniramine", "promethazine", "triprolidine", "dextromethorphan", "dihydrocodeine", "pentoxyverine", "pholcodine", "guaiphenesin", "Ipecacuanha", "senega and ammonia", "phenylephrine", "pseudoephedrine", "oxymetazoline", "xylometazoline", and "hedera helix (ivy leaf extract)". Calls with the "herbal preparations" substance code was also extracted and manually reviewed for inclusion to identify herbal preparations marketed for treatment of coughs and colds. We separately analysed data for sedating antihistamine/decongestant combinations because a product (brompheniramine/phenylephrine) was available from 2015 with dosing instructions for 2–5 year-old children for allergic rhinitis.[3]

We used the Joinpoint regression program (version 4.8.0.1) to evaluate changes in reporting over time (annual percentage change, APC), using the permutation test model selection method. All other analyses were conducted with R 4.1.0. *P* <0.05 was deemed statistically significant.

References

1. OTC cough and cold medicines: not for younger children. *React. Wkly.* 2012; 1416: 4.
2. Therapeutic Goods Administration. First-generation oral sedating antihistamines: do not use in children [media release]. 13 July 2022. https://www.tga.gov.au/news/safety-updates/first-generation-oral-sedating-antihistamines-do-not-use-children (viewed Feb 2023).
3. AusDI [internet]. Health Communication Network; c2023. Product Infortmation: Dimetapp Kids 2 to 5 Years Allergic Rhinitis Colour Free. Available from https://ausdi-hcn-com-au.productInformation.hcn?file=p03718 (updated 2016, viewed Feb 2023)

Supplementary results

Table 1. Exposures of childen under six years of age to cough and cold products reported to the New South Wales Poisons Information Centre, 2010–2021*

Preparation type	2010	2011	2012	2013	2014	2015	2016	2017	2018	2019	2020	2021	Total
Cough suppressant	112	117	95	66	49	49	48	42	48	65	31	34	756 (9.1%)
Cough suppressant combinations	152	159	147	82	41	38	19	22	18	9	12	15	714 (8.6%)
Decongestant	66	77	68	66	62	67	65	76	67	69	45	69	797 (9.6%)
Expectorant	67	61	37	32	22	21	18	12	13	18	20	10	331 (4.0%)
Herbals	27	29	40	49	104	162	156	148	175	242	175	300	1607 (19.3%)
Mucolytic	36	36	37	18	20	21	21	25	28	27	21	23	313 (3.8%)
Sedating antihistamine	165	153	159	135	123	124	128	90	90	118	112	122	1519 (18.2%)
Sedating antihistamine/ decongestant	267	236	222	122	93	99	142	117	111	96	73	41	1619 (19.4%)
Sedating antihistamine/ decongestant/cough suppressant	5	5	1	0	0	1	1	0	1	7	12	8	41 (< 1%)
Unspecified	194	165	155	69	4	10	6	5	3	8	6	4	630 (7.6%)

Table 2. Characteristics of the 8327 cough and cold medication exposures of children under 6 years of age reported to the NSWPIC, 2010–2021

Characteristic	Value
Age (months), median (interquartile range)*	32 (24–42)
Sex	
Boys	4249 (51.0%)
Girls	3817 (45.8%)
Unknown	261 (3.1%)
Exposure type	
Accidental	4264 (51.2%)
Therapeutic error	3953 (47.5%)
Adverse reaction	76 (< 1%)
Other/undetermined	34 (< 1%)
Disposition	
Managed at home	7074 (85.0%)
Hospital management†	1020 (12.2%)
Other	153 (1.8%)
General practitioner‡	80 (1.0%)

* Based on 8061 cases for which exact age was recorded (96.8%); for 266 children, only age range was recorded.

† Call to NSWPIC was from a hospital, or NSWPIC referred the patient to hospital.

‡ Call to NSWPIC was from a general practice, or NSWPIC referred the patient to a general practitioner.

Table 3. Estimated changepoints and APCs for over-the-counter (OTC) cough and cold medication exposures (joinpoint regression)

Outcome	Changepoints	Segment	Annual percentage change (95% CI)	*P*
All OTC cough and cold medications	2014 (2012–2016)	Whole period	−6.3% (−10.3% to −2.0%)	0.004
		2010–2014	−17.3% (−26.5% to −6.9%)	0.007
		2014–2021	0.7% (−4.2% to 5.8%)	0.77
Products affected by the 2012 labelling change	2014 (2013–2016)	Whole period	−11.1% (−14.5% to −7.5%)	< 0.001
		2010–2014	−21.4% (−29.2% to −12.7%)	0.001
		2014–2021	−4.6% (−8.8% to −0.3%)	0.040
Herbal cough and cold medications	2015 (2013–2018)	Whole period	25.1% (16.0% to 34.9%)	< 0.001
		2010–2015	43.9% (24.0% to 66.9%)	0.001
		2015–2021	11.3% (−0.6% to 24.5%)	0.06
Sedating antihistamine/ decongestant combination	2014 (2012–2016)	Whole period	−15.8% (−26.2% to −3.8%)	0.011
		2010–2014	−23.7% (−39.5% to −3.6%)	0.032
	2018 (2015–2019)	2014–2018	5.8% (−26.8% to 53.0%)	0.69
		2018–2021	−29.2% (−51.0% to 2.4%)	0.06
Sedating antihistamines (alone)	2018 (2016–2019)	Whole period	−2.8% (−6.4% to 1%)	0.15
		2010–2018	−6.8% (−9.8% to −3.8%)	0.001
		2018–2021	9% (−5.8% to 26.3%)	0.20

Figure 1. Numbers of calls to NSWPIC by year ("observed"; red squares) and change points in trend identified by joinpoint regression analysis

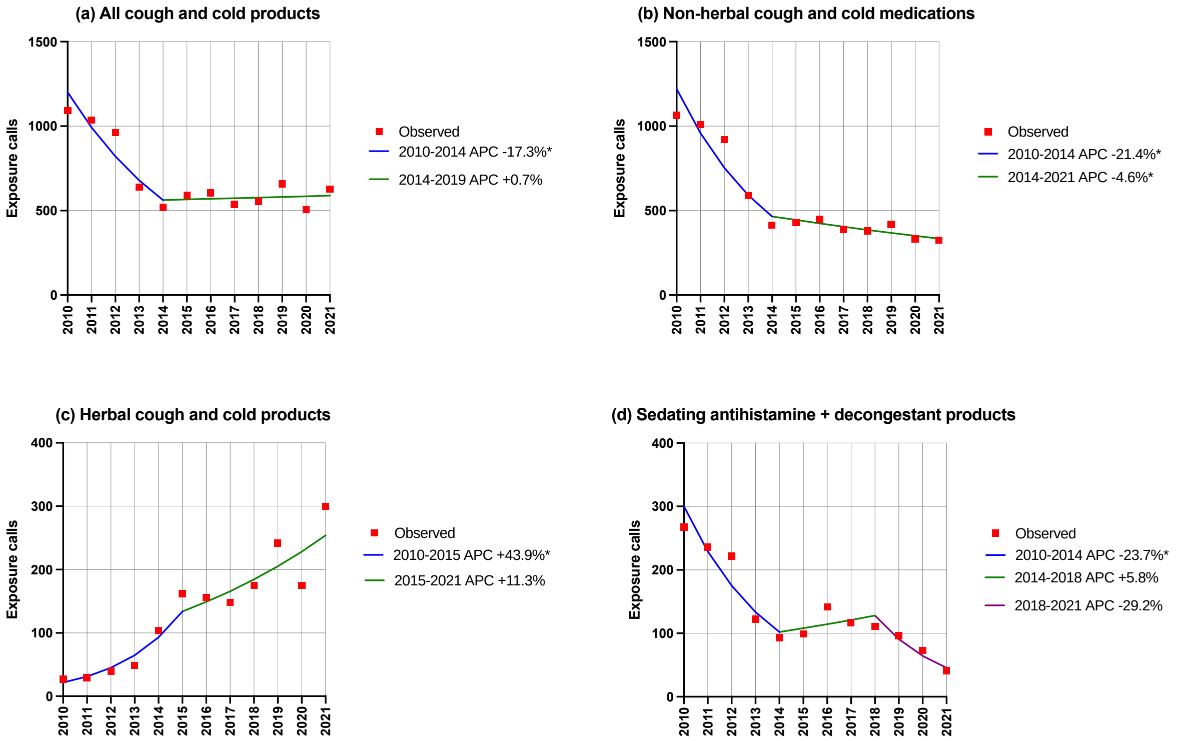

* $P < 0.05$.
